# Ignorance, Fatal Effects of

**Published:** 1854-02

**Authors:** 


### IGNORANCE.

The following article shows the fatal result of taking a common and simple medicine on one's own responsibility. Thousands of persons, especially in cities, in order to avoid doing with physicians, as it is termed, will purchase a patent medicine and take five times as much physic in a week as a scientific practitioner would have administered in a month—the labels often running "from one to two table-spoons three or four times a day:"

Death from an over-dose of salts.—Coroner O'Donnell yesterday held an inquest, at No. 325 Spring st., upon the body of Mary Flanagan, a native of Ireland, 62 years of age, who died suddenly, shortly after taking a quarter of a pound of Epsom salts. She had been unwell for some hours, when her daughter, Mrs. Catherine Sully, happened to come in, and recommended a dose of salts as a remedy. A quarter of a pound was immediately procured and administered by the daughter to her mother, who died about an hour afterwards. One witness testified that the quantity taken was sufficient for four doses for a person of the age of the deceased. A post-mortem examination of the body was made by a physician, who gave it as his opinion that death was caused by disease of the heart, aggravated by an over-dose of Epsom salts, and the jury rendered a verdict to that effect. The deceased had been in the country but four months.

